# Supplementary figures and images for: Health-Related Field-Based Fitness Tests: Normative Values for Italian Primary School Children
Source: J Funct Morphol Kinesiol. 2024 Oct 9;9(4):190. doi: 10.3390/jfmk9040190 (PMC11503293; doi:10.3390/jfmk9040190)

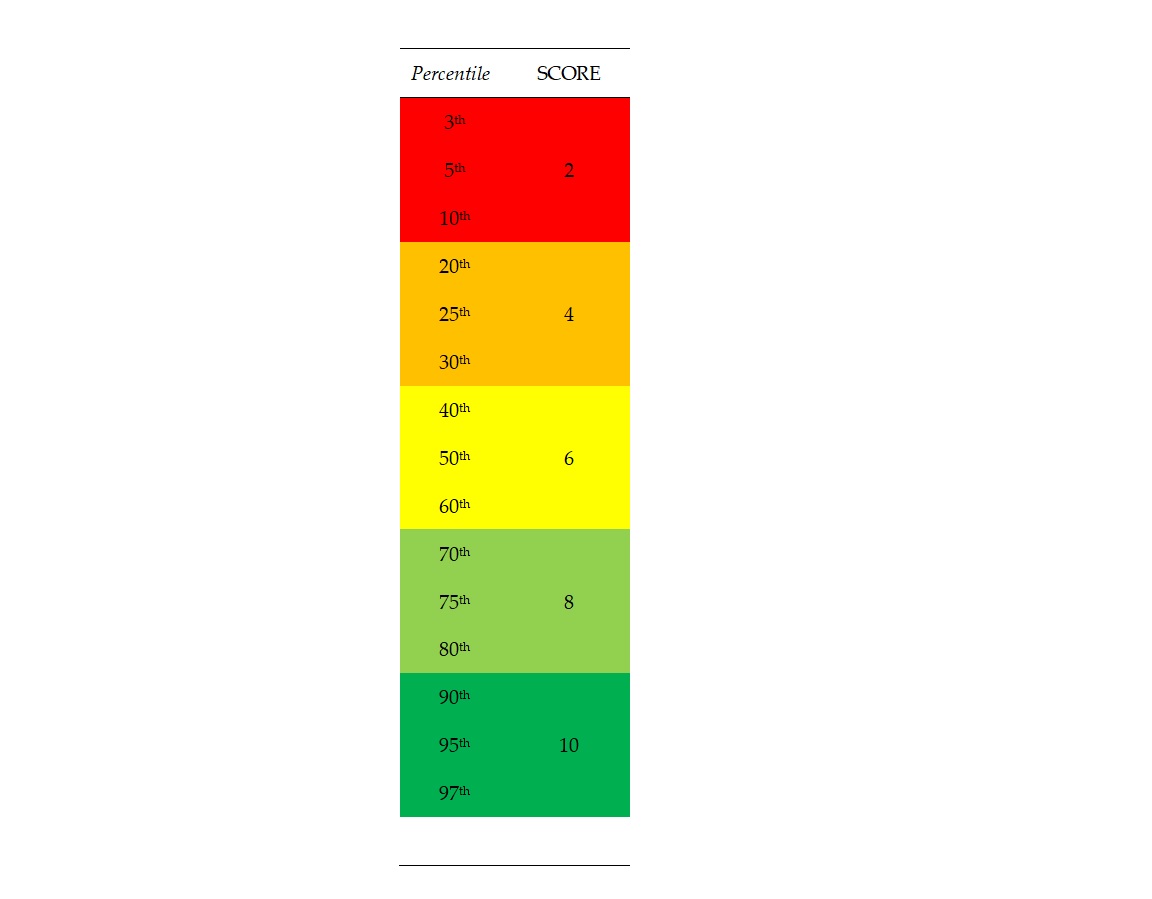

Supplement: Supplementary file 1 [file jfmk-09-00190-s001.zip › Figure S2 Physical Fitness-Motor Competence Performance Index (PF-MC PI) calculation ac-cording to percentiles.jpg]

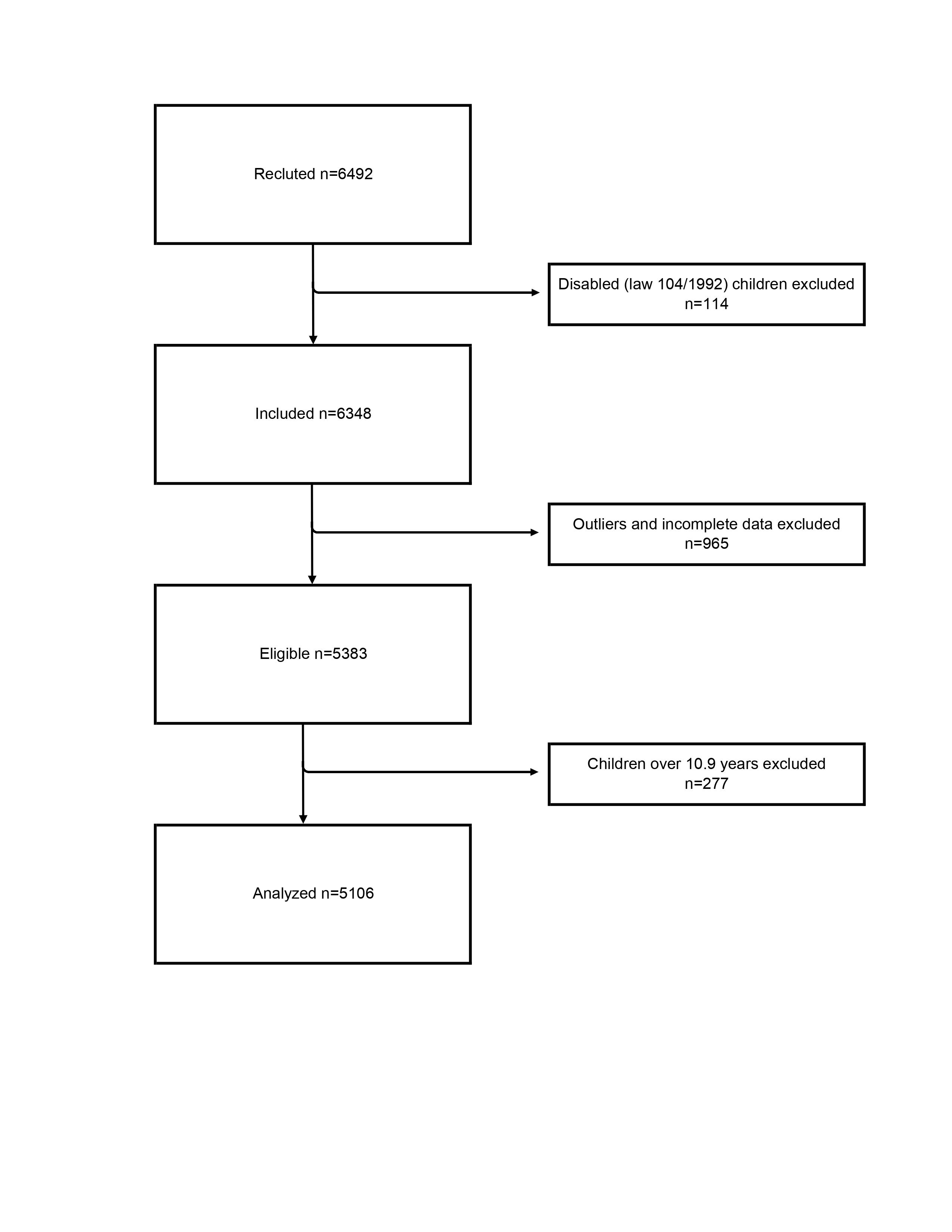

Supplement: Supplementary file 1 [file jfmk-09-00190-s001.zip › Figure S1 Flowchart of the enrollment process of the study sample.jpg]
